# The experience of bodily image for patients with left ventricular assist device

**DOI:** 10.3389/fpsyt.2024.1484428

**Published:** 2025-01-23

**Authors:** Irena Milaniak, Emilia Witkowska, Marta Cebula, Paulina Tomsia, Grzegorz Wasilewski, Izabela Górkiewicz-Kot, Sylwia Wiśniowska-Śmiałek, Michał Kaleta, Karol Wierzbicki

**Affiliations:** ^1^ Andrzej Frycz Modrzewski Krakow University, Faculty of Health Sciences, Krakow, Poland; ^2^ Clinical Department of Cardiovascular Surgery and Transplantology, Krakow Specialist Hospital of Saint John Paul II, Krakow, Poland; ^3^ Jagiellonian University Medical College, Krakow, Poland

**Keywords:** left ventricular assistance device, body image, patient, anxiety, depression

## Abstract

**Introduction:**

Mechanical Cardiac Support and Left Ventricular Assist Devices (LVAD) have been demonstrated to prolong Heart Failure patients' survival and improve their quality of life. LVAD implantation has a considerable effect on patients' body image. Patients find it hard to accept the device as an extension of their body, especially the driveline. The study aimed to examine the relationships between anxiety and depressive symptoms, acceptance of illness, beliefs about pain control, and quality of life with body image among LVAD-implanted patients.

**Methods:**

The cross-sectional study included 54 conveniently recruited patients who completed the Body Image Scale, SF-12, Acceptance of Illness Scale, Beliefs about Pain Control Questionnaire, NRS, HADS, and demographic and clinical data. Multiple regression analyses examined the associations between the research variable.

**Results:**

The mean Age of the participants was 59.64 (SD=9.63), and 96.3% were men. The mean scores were: Body Image Scale – 28.33 (SD=5,91); Acceptance of Illness Scale – 25.51 (SD=5.92); beliefs about pain control: internal factors – 15.85 (SD=4.96), the influence of physicians – 17.57 (SD=3.15), random events – 14.37(SD=3.32), NRS=2.80(SD=1.86), HADS anxiety – 5.33 (SD=4.12), HADS depression – 4.66 (SD=3.10), SF12MCS-45.49 (SD=6.48), SF-12PCS-41,33 (SD=6.48). The presence of anxiety and depressive symptoms and complications after LVAD significantly predicted low body image concerns.

**Discussion:**

Healthcare professionals should be aware of challenges regarding body image faced by LVAD-implanted patients and address related factors, especially anxiety and depression.

## 1 Introduction

Heart Failure (HF) is a growing health and economic burden for the whole world, in large part because of the aging population (1). A subset of patients with chronic heart failure will continue to deteriorate and experience persistently severe symptoms despite receiving the highest level of recommended medical treatment. HF hospitalization rates in Poland are among the highest in Europe, at 547:100,000 inhabitants (2).

Durable Left Ventricular Assist Devices (LVADs) should be considered in selected patients with NYHA class IV symptoms who are deemed dependent on IV inotropes or temporary Mechanical Cardiac Support (MCS) (3). The magnitude of the survival benefit for durable LVAD support in advanced NYHA class IV patients has progressively improved, with two-year survival greater than 80% in recent trials with newer generation LVADs, which approach early survival after cardiac transplantation (4, 5). The 2020 INTERMACS (Interagency Registry for Mechanically Assisted Circulatory Support) report showed that 87.6% of recent durable LVAD recipients were categorized as INTERMACS 1 to 3 before implant surgery. It also showed improved mean survival, greater than four years for the destination LVAD cohort and greater than five years for bridge-to-transplant patients (6).

Durable LVAD support has also achieved impressive functional and quality of life (QOL) improvements in multiple trials. However, patients remaining tethered to external electrical power supplies via a percutaneous lead can limit this improvement (7, 8). Most patients require rehospitalization within the first year post-implant (9). These factors emphasize the need for a thorough evaluation and patient education before the decision to proceed with the treatment. Appropriate patient selection benefits from review by a multidisciplinary team that typically includes an HF cardiologist, surgeon, social worker, nurse, psychologist, pharmacist, dietician, and palliative medicine specialist (10).

An MCS, such as an LVAD, is a mechanical pump surgically implanted into the patient’s chest to support heart function and blood flow. An LVAD consists of an inflow and outflow cannula, a pump, a driveline, a system controller, and a power source. The controller sends power and operating signals through the driveline. The driveline is connected to the pump on one end, exits the patient’s body at the chest or abdomen region, and connects to the external controller on the other (11). The LVAD controller is connected to two external rechargeable battery packs that the person carries (12). These bulky and heavy external components are typically held in a bag or harness worn over the shoulders. All equipment weighs about 2.5 kg.

As the number of people using LVAD increases, research on the impact of LVAD use on psychosocial well-being has emerged. People with long-term physical health conditions are two to three times more likely to experience mental health issues than the general population (13, 14).

The review builds on Thomas F. Cash’s definition of body image as a multidimensional construct encompassing self-perceptions and attitudes regarding physical appearance (15). Consistent with Cash’s definition, attitudinal body image (BI) consists of at least two dimensions: (1) evaluation/affect, which includes body-image appraisals and satisfaction/dissatisfaction, and (2) investment, such as the salience, centrality, or extent of cognitive-behavioral emphasis on one’s appearance (15, 16). Negative BI is associated with depressive symptoms, low body esteem, and poorer quality of life (17, 18). Patients who are required to wear some external medical equipment, i.e., Stoma bags, ICD, experience significant distress accepting/adapting to an altered body (19–22). An LVAD can have considerable effects on a patient’s sense of self and perception of BI, resulting in profound psychological sequelae for some patients and their families (23). Disturbance in bodily experience (BE) can result from ventricular assist device (VAD) implantation. BE encompasses all cognitive and emotional processes associated with an individual’s perception of their own body (24). It is not uncommon that psychiatric symptoms, such as depression, anxiety, and posttraumatic stress disorders, are underdiagnosed and may be undertreated in patients with LVAD, which can affect their overall QOL and survival (23, 25, 26).

The implantation of LVAD has been shown to impact patients’ body image significantly. Patients find it difficult to accept the device as part of their body; they perceive their body differently and may even feel disgusted by it. In small studies, almost half of patients reported that the LVAD device had a negative impact on their self-image and sexual function (27). The study, led by Charton M. et al., analyzed the psychological outcomes of LVAD implantation in 494 end-stage heart failure patients. They discovered a 2% risk of attempted or completed suicide in LVAD recipients, which is higher than in the general population or those with other chronic diseases in France. Several potential factors, such as changes in body image, inability to return to full-time employment, feeling like a burden to caregivers, and increased dependence on the medical team, could contribute to the development of psychiatric symptoms in LVAD recipients (28). This study aimed to explore the relationships between anxiety and depressive symptoms, acceptance of illness, beliefs about pain control, and quality of life with body image among LVAD-implanted patients.

## 2 Materials and methods

### 2.1 Study design

Cross-sectional study. The study protocol complied with the Declaration of Helsinki and was approved by the Bioethics Committee KBKA/23/O/2021.

### 2.2 Setting

The study was conducted at the Department of Cardiovascular Surgery and Transplantology of the John Paul II Specialist Hospital in Krakow from 1.01.2022 to 31.12.2022. The department has 27 beds and is dedicated to patients with advanced heart failure eligible for surgical treatment: heart transplantation or left ventricular assist device implantation. The department has experience in the surgical treatment of heart failure: heart transplantation (since 1988), cf-LVAD (2015). The center is a participant in the PCHF-VAD registry (29). Annually, 20 heart transplant procedures and 20 procedures of mechanical circulatory support implantation are performed at the center.

### 2.3 Participants

Patients meeting the following criteria were enrolled in the study: Age > 18 years old, good cognitive status, co-residence with the patient/caregiver, first hospitalization after LVAD implantation completed, and informed consent to participate in the study.

The exclusion criteria included Age < 18 years, poor cognitive health, no caregiver, first hospitalization after LVAD implantation, and participating in other clinical trials. Fifty-four patients were enrolled in the study, and the participants of the project were “Determinants of the quality of life of the caregiver and patient with mechanical circulatory system support.”

### 2.4 Instruments and measures

1. The SF-12 is a short-form health status survey with 12 questions. SF-12 includes Physical Component Summary (PCS) and Mental Component Summary (MCS). Calculation of scores for the eight scales is performed using the transformed scores (range: 0-100), and summary measures are standardized to produce a mean of 50 with a standard deviation of 10 for the United States (US) population (norm-based scoring) (30). A License for using the SF-12v2 was acquired from QualityMetric Incorporated (QM0557070, August 2021). Test reliability of the SF-12 for the research was measured by the Cronbach Alfa coefficient, which was for PCS – 0.77 and MCS – 0.73.
2. The Hospital Anxiety Depression Scale (HADS) is a generic screening instrument that measures symptoms of anxiety and depression (31, 32). The HADS scale consists of two independent subscales measuring the level of anxiety and severity of depression. Each subscale contains 7 statements regarding the subject’s current state, which can be rated from 0 to 3 points. The result for each subscale falls between 0 and 21 points; higher scores indicate more severe symptoms of anxiety and depression. A score between 0 and 7 indicates a standard value, a score between 8–10 points suggest the ceiling, whereas a score between 11 and 21 is considered abnormal. Test reliability of the HAD-Scale for own research measured by the coefficient Cronbach Alfa was 0.76 for anxiety and 0.74 for depression.
3. The Body Image Scale (BIS) consists of 11 questions. The questionnaire was adopted from the Body Image Scale and Confidence in LVAD technology (27). The total score varied from 0 to 44 points. Higher BIS scores represented a more positive perception of body image. Test reliability of the Body Image Scale for the research, measured by the coefficient Cronbach Alfa, was 0.838
4. Acceptance of Illness Scale (AIS) The AIS scale consists of 8 statements describing the consequences of poor health in assessing limitations imposed by the illness, lack of self-sufficiency, a sense of dependence on others, and reduced self-esteem. In each statement, the examined patient determines their current state on a 5-degree scale. Strong agreement (score 1) expresses poor adaptation to the illness, while a strong disagreement (score 5) means acceptance of the disease. The general measure of acceptance of illness is the sum of all points, and its range is 8–40 points. A low score means no acceptance and adaptation to the disease and a strong sense of mental discomfort; a high score indicates acceptance of one’s medical condition and is manifested by the lack of negative emotions associated with the illness (33). Test reliability of the AIS-Scale for the research, measured by the coefficient Cronbach Alfa, was 0.81
5. Beliefs about Pain Control Questionnaire (BPCQ). The BPCQ assessment, measuring the strength of individual beliefs about pain control, consists of 13 statements that make up three factors of pain control: internal factors (a belief in pain control personally), influence of physicians (a belief that others control pain); random events (a belief that there is no self-influence on pain control). The patient should assess the statements in the questionnaire on a six-point Likert scale, where 1 means “no, I completely do not agree” and 6 indicates “yes, I agree.” The sum of the results is calculated separately. The range of points possible to obtain is from 5 to 30, in external control and from 4 to 24 in the influence of physicians and random events. The higher the score of a given area, the stronger the belief that the patient can control pain through the strength of a given factor (33). The test reliability of the BPCQ scale for the research, measured by the Cronbach Alfa coefficient, was 0.74.
6. A numerical rating scale (NRS) rates the patient’s pain from 0–10, where 0 is no pain, and 10 is the worst pain imaginable.
7. Demographic variables included Age, gender, place of residence, professional activity, education, and marital status. Clinical data included the duration of therapy, type of device, patient body weight, and complications related to LVAD (driveline infection, other complications like bleeding thromboembolic events, ischemic stroke, right ventricular failure).

### 2.5 Statistical analysis

Statistical analysis was performed in R, version 4.4.0. The analysis of quantitative variables (i.e., expressed in numbers) was performed by calculating descriptive statistics such as mean, standard deviations, median, quartiles, and minimum and maximum. The analysis of qualitative variables (i.e., not expressed in numbers) was carried out by calculating the absolute frequencies and percentages of all values that these variables could assume.

A univariate analysis of the impact of potential predictors on a dichotomous variable (i.e., taking on only two possible values) was performed using logistic regression. The results are presented as OR parameters (odds ratio) with 95% confidence intervals. A significance level of 0.05 was adopted in the analysis.

## 3 Results

### 3.1 Patient and caregiver demographics

The dominant gender among patients was male (96.3%). The median Age of the patients was 61 years. The mean time since LVAD implantation was 3.11 years (SD 1.84). The type of device implanted was HeartMate II (Thoratec Corporation, Pleasanton, CA, USA) in 37 patients and HeartWare in 17 (HeartWare, Framingham, MA, USA). The mean duration of mechanical support was 3,11 ± 1.84 years. Body weight ranged from 57.2-130 kg, mean body weight was 86.33 kg. DLI occurred in 31 (57.41%) of the subjects and other complications in 10 (18.52%). Demographic and clinical data are presented in **Table 1**.

**Table 1:** Sample demographic and clinical characteristic.

Variable		(N=54)
Age [years]	Mean (SD)	59,65 (9,63)
	Median (quartiles)	61 (55,25-67)
	Range	29-74
	n	54
Gender	Men	52 (96,30%)
	Women	2 (3,70%)
Place of living	City	20 (37,04%)
	Village	34 (62,96%)
Duration of LVAD therapy [years]	Mean (SD)	3,11 (1,84)
	Median (quartiles)	3,18 (1,56-4,76)
	Range	0,3-7,16
	n	54
Activity (employment)	Inactive professionally	48 (88,89%)
	Active professionally	6 (11,11%)
Education	Primary	2 (3,70%)
	Secondary	44 (81,48%)
	High	8 (14,81%)
Marital status	Single	2 (3,70%)
	Married	51 (94,44%)
	Divorced	1 (1,85%)
	
Kind of LVAD	HM	37 (68,52%)
	HW	17 (31,48%)
Pain (NRS)	Mean (SD)	2,8 (1,87)
	Median (quartiles)	3 (1-4)
	Range	0-8
	n	54
Body weight [kg]	Mean (SD)	86,33 (16,2)
	Median (quartiles)	85,65 (76-94,83)
	Range	57,2-130
	n	54
Complications	Without	13 (24,07%)

SD, standard deviation; n, number; LVAD, Left Ventricular Assist Device; HM, HeartMate; HW, HeartWare; NRS, Numerical Rating Scale; kg, kilogram.

### 3.2 Body image perception

The mean score on the Body Image Scale was 28.33 (SD 5.91). A high level of body perception was declared by 77.78% of respondents. Analyzing the questions regarding the acceptance of the device, the average answer was 2.86 ± 0.12; for statements regarding body image perception, the average answer was 2.49 ± 0.22. **Tables 2**, **3**.

**Table 2:** The results of standardized tools.

Tool		Mean	SD	Median	Min	Max	Q1	Q3
Body Image Scale		28,33	5,91	30,00	14,00	43,00	23,00	33,00
	Acceptance of Illness Scale	25,52	5,92	25.00	10.00	40.00	23.00	29.00
Hospital Anxiety Depression Scale	Anxiety	5,33	4,12	5,00	0,00	21,00	3,00	7,00
	Depression	4,67	3,10	4,00	0,00	10,00	2,00	7,00
							
The Beliefs about Pain Control Questionnaire	Internal factors	15,85	4,97	14,00	7,00	30,00	13,00	18,75
	Influence of physicians	17,57	3,15	17,00	11,00	24,00	16,00	20,00
	Random events	14,37	3,33	14,00	8,00	24,00	12,25	15,75
SF-12	PCS	41,33	6,49	41,87	28,19	57,10	37,18	45,29
	MCS	45,49	6,67	45,58	33,35	64,27	41,21	50,06

SD, standard deviation; Q1, lower quartile; Q3, upper quartile; SF, Short Form; PCS, physical component score; MCS, mental component score.

**Table 3:** Distribution of results of the body image scale.

Range of points	Interpretation	n	%
0-22	Low body image acceptance	12	22,22%
23-44	High body image acceptance	42	77,78%

### 3.3 Illness acceptance

The average sum of all Acceptance of Illness Scale scores for the study group was 25.52. The results obtained are within the average range for chronically ill people. The mean of the results of individual scale items was 3.19 ± 0.19. Analyzing the individual statements of the scale, it was found that the respondents obtained the lowest average results for the question relating to the self-sufficiency of the respondents (2.63 ± 0.96). However, the highest average results were obtained for the question regarding the feeling of embarrassment in people staying with the respondents (3.79 ± 1.08). **Table 2**.

### 3.4 Anxiety and depression

The mean scores for anxiety and depression amounted to, respectively, 5,33 ± 4,12 and 4,67 ± 3,10. 13% (n=7) of patients presented mild to severe symptoms of depression, and 18,5% (n=10) of patients presented mild to severe symptoms of anxiety. **Table 2**.

### 3.5 Pain assessment

The average pain intensity on the Numeric Rating Scale (NRS) score was assessed at level 3 (min -0, max - 8).

Beliefs about Pain Control Questionnaire (BPCQ).

The analysis of the strength of individual beliefs about pain control showed that physicians had the most significant influence on the pain perception of the subjects (mean 17.57 ± 3.15). Internal factors (IF) (mean 15.85, ± 4.97) and the least influence were random events (RE) (mean 14.37 ± 3.33), **Table 2**. In the typology based on the division of results at the median point, the type increasing the influence of doctors dominated (low IF, high D, low AI).

### 3.6 Relationships among variables

The correlation analysis showed that body image perception was positively related to the level of illness acceptance, pain control (internal), and quality of life in the physical domain. In other words, participants with high levels of illness acceptance, quality of life in the physical domain, and internal pain control have a better perception of their body image. Negative correlations were found with pain intensity, depression, and anxiety. In other words, participants with higher intensity of pain, anxiety, and depressive symptoms have lower body image. The highest correlation was between body image and depression and anxiety (r=-0.56, P< and r=-0.51, p<) (**Table 4**).

**Table 4:** Correlation matrix.

		1.	2.	3.	4.	5.	6.	7.	8.	9.	10.	11.	12.
1. Age	Pearson correlation Bilateral significance	1											
											
2. Body Image Scale	Pearson correlation Bilateral significance	.199	1										
		.149											
3. AIS	Pearson correlation Bilateral significance	-.099	.366^**^	1									
		.478	.007										
4. IF BPCQ	Pearson correlation Bilateral significance	.039	.293^*^	-.111	1								
		.779	.032	.423									
5. IP BPCQ	Pearson correlation Bilateral significance	.163	-.078	-.307^*^	.160	1							
		.238	.574	.024	.249								
6. RE BPCQ	Pearson correlation Bilateral significance	-.044	-.066	-.416^**^	.216	.341^*^	1						
		.751	.636	.002	.117	.012							
7. NRS	Pearson correlation Bilateral significance	-.315^*^	-.399^**^	-.152	-.152	.446^**^	.158	1					
		.020	.003	.272	.273	<.001	.253						
8. Time since LVAD	Pearson correlation Bilateral significance	.056	-.158	-.223	.158	.275^*^	-.058	.051	1				
		.687	.255	.105	.253	.044	.674	.712					
9. Anxiety	Pearson correlation Bilateral significance	-.014	-.511^**^	-.229	-.460^**^	.272^*^	-.012	.573^**^	.091	1			
		.922	<.001	.096	<.001	.046	.932	<.001	.511				
10. Depression	Pearson correlation Bilateral significance	-.029	-.556^**^	-.143	-.336^*^	.182	-.125	.457^**^	.119	.641^**^	1		
		.837	<.001	.301	.013	.188	.368	<.001	.392	<.001			
11. PCS	Pearson correlation Bilateral significance	-.082	.196	.255	-.135	-.139	-.149	-.115	.362^**^	-.072	-.168	1	
		.556	.156	.063	.329	.316	.284	.409	.007	.607	.224		
12. MCS	Pearson correlation Bilateral significance	.042	.421^**^	.279^*^	.164	-.369^**^	.140	-.388^**^	-.416^**^	-.414^**^	-.439^**^	-.129	1
		.763	.002	.041	.237	.006	.314	.004	.002	.002	<.001	.353	

*Correlation significant at the 0.05 level (bilateral) ** Correlation significant at the 0.01 level (bilateral), AIS, Acceptance Illness Scale; BPCQ, Beliefs about Pain Control Questionnaire; IF, Internal factors; IP, Influence of physicians; RE, Random events; PCS, physical component score; MCS, mental component score; NRS, Numerical Rating Scale; LVAD, Left Ventricular Assist Device.

### 3.7 Predictors of body image perception

Logistic regression models for each considered variable showed that each subsequent “anxiety point” increases the chances of low body image perception by 20.3% (OR=1.203). Each subsequent “depression point” increases the chances of body image perception by 41.7% (OR=1.417). The occurrence of complications other than DLI increases the odds of low body image perception 12 times (OR=12) compared to no complications (**Table 5**).

**Table 5:** Predictors of body acceptance.

Variable		N	N	OR	95%CI		p
Age [years]		-	-	0,962	0,903	1,025	0,23
Kind of device	HM	37	9	1	ref.		
	HW	17	3	0,667	0,156	2,858	0,585
Durable of therapy [years		-	-	1,341	0,93	1,934	0,117
PCS		-	-	0,959	0,867	1,06	0,411
MCS		-	-	0,9	0,805	1,006	0,064
AIS		-	-	0,953	0,853	1,065	0,399
Anxiety		-	-	1,203	1,019	1,421	0,029 *
Depression		-	-	1,417	1,112	1,805	0,005 *
Pain (NRS)		-	-	1,347	0,939	1,931	0,106
Internal factors (BPCQ)		-	-	0,916	0,787	1,067	0,26
Influence of physicians (BPCQ)		-	-	1,057	0,862	1,296	0,593
Random events (BPCQ)		-	-	0,976	0,801	1,189	0,808
Body Weight [kg]		-	-	0,991	0,951	1,032	0,656
Complication	Without	13	1	1	ref.		
	Driveline infection	31	6	2,88	0,311	26,679	0,352
	Other complications	10	5	12	1,103	130,58	0,041 *

OR, odds ratio; CI, confidence interval; AIS, Acceptance Illness Scale; BPCQ, Beliefs about Pain Control Questionnaire; PCS, physical component score; MCS, mental component score; NRS, Numerical Rating Scale; LVAD, Left Ventricular Assist Device. Bold values present significant values.

## 4 Discussion

This study is one of the few studies assessing body perception in the context of LVAD therapy and factors influencing body perception, and it is the first study in Poland.

Rhoades BD. et al. conducted a systematic review to summarize the factors influencing left ventricular assist device adaptation. Adapting to an LVAD presents unique challenges attributed to the external device components (e.g., managing the batteries and external components, limitations in bathing and swimming, alterations in body image, effects on intimacy, and emotional distress). Among numerous factors, alterations in their body image were one of the factors that influenced living with LVAD (34). Our results showed that 77.78% of patients living with LVAD experienced high body acceptance. A previous study found that patients with LVAD are satisfied with this kind of device and tend to accept it as a part of their bodies and lives (35, 36). Melnikov S. et al. also showed higher body acceptance (27). On the contrary, Tosto C. et al. showed that patients reported significant devices-related distress and low body image concern (37). Furthermore, Marcuccilli L. et al. described a woman who struggled to adapt to an altered body image caused by the external components of the LVAD system (38). Çamlica T. et al. described the adaptation process of patients with LVAD using the Roy Adaptation Model. They diagnosed disturbance in body image, depressive symptoms, aggression, and social isolation (39). Inyom C. et al. identified that patients emphasized burdens from their devices, such as weight and handling, limitations in their physical ability, reduced social interactions, and reduction in sexual activity and performance (40).

Disturbance in-body experience is a potential consequence of LVAD therapy, and it is known that even relatively small disfigurements can have a significant impact on psychosocial outcomes. Our bivariate analysis showed the correlations between body image and level of acceptance of illness, level of internal pain control, level of pain, PCS domain of QOL, and depression and anxiety. Depression and anxiety other than driveline infection complications after LVAD implantation revealed the predictors of low body image. Other complications in our study include right ventricular failure, rehospitalization, and ischemic stroke events. The large study made by Makuel LM et al. shows that adverse events, readmission, stroke, and gastrointestinal bleeding after left ventricular assist device implantation are linked to psychosocial risk (41). In their study, Tosto C. et al. also revealed a strong correlation between body image and depression and anxiety (37). Melnikow S. et al. l also found a relationship between anxiety, depression, and body image. They also stated that depression alone, or depression combined with anxiety, moderated the relationships between body image and personal well-being (42). In the other study, Melnikov S. et al. revealed that sexual functioning and device technology confidence significantly predicted body image (27). Richter F. et al. showed that body image disturbances were more common in women but not at a high level. Moreover, these disturbances decreased with time after LVAD implantation (24). Our study did not confirm this relationship.

Marcuccilli L. and all, in their study, drew attention to how patients manage threats to self-concept to live with such a device safely. They concluded that participants accepted the LVAD as necessary to live: “Having an LVAD means living.” On the other hand, patients desired to be normal in public because their appearance was ‘shocking’ to others (38). In our study, when analyzing the Body Image scale separately, we observed that the environment accepted patients and did not feel that they were disfigured. These results also corresponded with illness acceptance, when patients achieved higher results in the question about the embarrassment of people staying with the respondents.

In our study, body image influences the physical component of quality of life and internal pain control. Levelink M and Levke Bru¨tt A. revealed in their systematic review that emotional reactivity influences the quality of live patients with LVAD. One of the factors is body image, which is associated with the prospect of spending the rest of life with the LVAD and associated limitations (e.g., first look in the mirror) (43).

Rapelli G. et al., in their qualitative study, revealed the embodying process among LVAD recipients. Patients and their caregivers began considering the device and the person as one (44). These results suggest that patients accept the device and have no problem with body image.

The durability of the device may play a role in developing anxiety and depression, and the main indications for mechanical circulatory support bridge to transplantation or destination therapy. Further, creating a new lifestyle to be able to daily manage the device and learning to live with physical limitations and a changed body image are also emotional challenges for LVAD patients (24, 36, 38). Patients positively evaluate their experience in a short period after implantation and are less satisfied with durable treatment due to worsening physical functioning (37). This is connected with striving for normality. Normalcy and safety represent overarching themes to balance daily living with a ventricular assist device and require health-related functional, social, and mental needs (45).

Study limitation

This study is the first to report the assessment of body image and factors that may influence body image in the LVAD population in Poland. The study limitations apply to the small number of participants, and generalizing across populations would not be warranted. Furthermore, the population was selected for the study “Determinants of the quality of life of the caregiver and patient with mechanical circulatory system support.” Most of the individuals in the present sample were men. Among women, the relationships between the variables examined may be different. Another restriction in the sample that affects the interpretation of the results is a specific sample. Patients after the first hospitalization, with poor cognitive status and no caregiver, were excluded. All these aspects that seem like negative determinants of health are excluded and probably affect the results. The multicenter study allows quicker recruitment of the necessary number of patients, more precise results that are more convincing, and whose acceptance is higher; furthermore, the patient sample of the multicenter is supposed to be representative. Secondly, the data collection was limited to one cardiac surgery and transplantology clinic.

## 5 Conclusions

This study shows that body image is associated with anxiety, depression, and complications after LVAD implantation. Other researchers confirmed these results. The results highlight the importance of raising the professional staff on the issue of LVAD implantation’s effect on body image. It is recommended to screen for disruption in body image during follow-ups, particularly among at-risk groups exhibiting symptoms of depression and anxiety, to ensure timely and targeted psychological support. Also, it seems to be important to screen for body image disturbances or difficulties preoperatively, as they can exacerbate after LVAD.

## Data Availability

The raw data supporting the conclusions of this article will be made available by the authors, without undue reservation.
